# Studies of inactivation mechanism of non-enveloped icosahedral virus by a visible ultrashort pulsed laser

**DOI:** 10.1186/1743-422X-11-20

**Published:** 2014-02-05

**Authors:** Shaw-Wei D Tsen, David H Kingsley, Christian Poweleit, Samuel Achilefu, Douglas S Soroka, TC Wu, Kong-Thon Tsen

**Affiliations:** 1 Department of Radiology, Washington University School of Medicine, St. Louis, Missouri 63110, USA; 2 US Department of Agriculture, Agricultural Research Service, Food Safety and Intervention Technologies Research Unit, James W. W. Baker Center, Delaware State University, Dover, Delaware 19901, USA; 3 Department of Physics, Arizona State University, Tempe, Arizona 85287, USA; 4 Department of Biochemistry and Molecular Biophysics, Washington University School of Medicine, St. Louis, Missouri 63110, USA; 5 Department of Biomedical Engineering, Washington University School of Medicine, St. Louis, Missouri 63110, USA; 6 U.S. Department of Agriculture, Agricultural Research Service, Eastern Regional Research Center, Wyndmoor, PA 19038, USA; 7 Departments of Pathology, Johns Hopkins School of Medicine, Baltimore, MD 21231, USA; 8 Departments of Oncology, Johns Hopkins School of Medicine, Baltimore, MD 21231, USA; 9 Departments of Obstetrics and Gynecology, Johns Hopkins School of Medicine, Baltimore, MD 21231, USA; 10 Departments of Molecular Microbiology and Immunology, Johns Hopkins School of Medicine, Baltimore, MD 21231, USA; 11 Center for Biophysics, Arizona State University, Tempe, Arizona 85287, USA

## Abstract

**Background:**

Low-power ultrashort pulsed (USP) lasers operating at wavelengths of 425 nm and near infrared region have been shown to effectively inactivate viruses such as human immunodeficiency virus (HIV), M13 bacteriophage, and murine cytomegalovirus (MCMV). It was shown previously that non-enveloped, helical viruses such as M13 bacteriophage, were inactivated by a USP laser through an impulsive stimulated Raman scattering (ISRS) process. Recently, enveloped virus like MCMV has been shown to be inactivated by a USP laser via protein aggregation induced by an ISRS process. However, the inactivation mechanism for a clinically important class of viruses – non-enveloped, icosahedral viruses remains unknown.

**Results and discussions:**

We have ruled out the following four possible inactivation mechanisms for non-enveloped, icosahedral viruses, namely, (1) inactivation due to ultraviolet C (UVC) photons produced by non-linear optical process of the intense, fundamental laser beam at 425 nm; (2) inactivation caused by thermal heating generated by the direct laser absorption/heating of the virion; (3) inactivation resulting from a one-photon absorption process via chromophores such as porphyrin molecules, or indicator dyes, potentially producing reactive oxygen or other species; (4) inactivation by the USP lasers in which the extremely intense laser pulse produces shock wave-like vibrations upon impact with the viral particle. We present data which support that the inactivation mechanism for non-enveloped, icosahedral viruses is the impulsive stimulated Raman scattering process. Real-time PCR experiments show that, within the amplicon size of 273 bp tested, there is no damage on the genome of MNV-1 caused by the USP laser irradiation.

**Conclusion:**

We conclude that our model non-enveloped virus, MNV-1, is inactivated by the ISRS process. These studies provide fundamental knowledge on photon-virus interactions on femtosecond time scales. From the analysis of the transmission electron microscope (TEM) images of viral particles before and after USP laser irradiation, the locations of weak structural links on the capsid of MNV-1 were revealed. This important information will greatly aid our understanding of the structure of non-enveloped, icosahedral viruses. We envision that this non-invasive, efficient viral eradication method will find applications in the disinfection of pharmaceuticals, biologicals and blood products in the near future.

## Background

Potential contamination of pharmaceuticals, biologicals, and uncooked foods with viruses is a critical problem. Conventional disinfection methods have potential serious side effects; for example, biochemical and pharmaceutical disinfection methods involve addition of potentially toxic or carcinogenic chemicals, such as detergents and photosensitizers which are impossible to remove completely after the treatments. In addition, the added chemicals may interact with the product itself, potentially altering its structure or function. Ionizing radiation such as ultraviolet and gamma rays can be used to sanitize foods and destroy viral pathogens in biologicals but amount of irradiation required for viruses is relatively high, potentially damaging biological products and cells and is less desirable for foods due to consumer concern. Microwave absorption method is not viable because water usually coexists with biological systems and water severely absorbs light in the microwave spectral range, leading to the heating effects.

An ultrashort pulsed (USP) laser technology has recently been developed to circumvent these difficulties [1-10]. The advantages of this novel technology are: (1) it is non-invasive; no foreign chemicals are added to the disinfection process, and therefore less concern for carcinogenic effects; (2) it does not use extremely high energy photons such as gamma or X-ray; as a result no covalent or ionic bonds are broken and less chance of creating new, potentially toxic materials; (3) it specifically targets the capsid of a virus; therefore, drug-resistant, mutated strains of the pathogens can also be killed by the technology; (4) it uses photons with wavelength transparent to water; consequently, in contrast to the microwave absorption method, it does not cause the heating effects.

Low-power ultrashort pulsed (USP) lasers operating at wavelengths of 425 nm and near infrared region have been shown [1-10] to effectively inactivate viruses such as human immunodeficiency virus (HIV), M13 bacteriophage, and murine cytomegalovirus (MCMV). It was shown previously [1-5] that non-enveloped, helical viruses such as M13 bacteriophage, were inactivated by a USP laser through an impulsive stimulated Raman scattering (ISRS) process. Recently, enveloped virus like MCMV has been shown [10] to be inactivated by a USP laser via protein aggregation induced by an ISRS process. However, the inactivation mechanism for a clinically important class of viruses – non-enveloped, icosahedral viruses remains unknown.

There are a variety of possible inactivation mechanisms for the non-enveloped, icosahedral viruses. These possibilities include: (i) inactivation due to ultraviolet C (UVC) photons produced by the intense laser beam through non-linear optical process; (ii) inactivation caused by thermal heating generated by the direct laser absorption/heating of the virion; (iii) inactivation resulting from a one-photon absorption process via chromophores such as porphyrin molecules, or indicator dyes, potentially producing reactive oxygen or other species; (iv) inactivation via an ISRS process.

We note that there is one more possibility of inactivation by the USP lasers in which the extremely intense laser pulse produces shock wave-like vibrations upon impact with the viral particle [11], leading to the viral inactivation. However, this possibility can be ruled out because the laser intensity employed in our current laser experiments is too low to activate such an effect.

In this paper, we report examination of these possible inactivation mechanisms for a non-enveloped, icosahedral virus, namely murine norovirus-1 (MNV-1), and present evidence that supports ISRS as the most likely inactivation mechanism by a visible USP laser. MNV-1 was chosen in this study because norovirus, which is highly contagious, is one of the leading viruses for food poisoning around the globe. This efficient, non-invasive approach for the eradication of non-enveloped, icosahedral viruses, when employed in a continuous, syringe-pumped configuration, can be applied to the disinfection of pathogens in the pharmaceutical processes and disinfection of blood components for blood transfusion.

## Materials and methods

### Virus stocks

Working stocks of MNV-1 were prepared using confluent monolayers of mouse monocyte/macrophage RAW 264.7 cells (American Type Culture Collection, Manassas, VA) cultured in high glucose Dulbecco’s modified eagle media (DMEM; Gibco-Invitrogen Co., Grand Island, NY) without indicator supplemented with 25 mM HEPES buffer, 10% fetal bovine serum (FBS; Gibco-Invitrogen), 2 mM Gluta-MAX-1 (Gibco-Invitrogen), 100 U of penicillin, and 100 μg/ml of streptomycin sulfate (Gibco-Invitrogen), essentially as described by Wobus et al. [12]. Partial purification of MNV-1 was performed essentially as described by Lou et al. [13]. MNV-1 stocks were treated with 10 μg/ml of DNAse for 1 hr followed by addition of 1% lauryl sarcosine and 10 mM EDTA. Virus was pelleted by centrifugation at 82,000 × *g* for 6 h at 4°C in a TH-660 rotor (Sorvall). The pellet was resuspended in PBS and further purified by centrifugation at 175,000 × g for 6 h at 4°C using a sucrose step gradient of 10, 20, 30, 40 and 45% sucrose. The pellet was resuspended in Earle’s balanced salt solution (Gibco-Invitrogen) and dialyzed against Tris-buffer saline (20 mM Tris-HCl, pH 7.6, 0.14 M NaCl).

MNV-1 samples were assayed using confluent 6-well dishes (Fisher Biotech, Fairlawn, NJ) inoculated with 0.5 ml, or ten-fold serial dilutions prepared in Earle’s balanced salt solution (EBSS; Life Sciences), for 2 h at 37°C followed by overlay with 2 ml of modified eagle media (Gibco-Invitrogen) containing 1.5% low melt agarose (Fisher Biotech) with 5% FBS, 2 mM Gluta-MAX-1, 100 U of penicillin and 100 μg/ml of streptomycin sulfate (Gibco-Invitrogen). After three days incubation, plaques were visualized by staining with 0.03% neutral red (Fisher Biotech) for 2 h at 37°C.

Because the 96-well tissue culture-treated flat bottom plates contain approximately 30,000 cells/well, depending upon the dilution of viral titer in the assaying process, the ratio of virus to cell can be 1.6 × 10^3^, 1.6 × 10^2^, 1.6 × 10^1^, 1.6, and 1.6 × 10^-1^.

### USP laser treatments

The excitation source employed in this work was a diode-pumped mode-locked Ti-sapphire laser. The laser produced a continuous train of 65 fs pulses at a repetition rate of 80 MHz. The output of the second harmonic generation (SHG) system of the Ti-sapphire laser was used to irradiate the sample. The excitation laser was chosen to operate at a wavelength of 425 nm and with an average power as specified. It had a pulse width of full-width at half maximum (FWHM) of about 100 fs. An achromatic lens of focus length 5 cm was used to focus the laser beam into the sample area. In order to facilitate the interaction of laser with MNV-1, the viral sample with a volume of 0.1 ml in buffer solution was placed inside a Pyrex cuvette with a micromagnet stirring bar and stationed above a magnetic stirrer so that virions would be forced to enter the laser-focused volume. The titer of MNV-1 samples was 5 × 10^7^ PFU/ml. The assays were performed on the laser-irradiated samples after proper dilution. The typical exposure time of the sample to laser irradiation was about 2 h. All the experimental results reported here were obtained at T = 25°C and with the single-laser-beam excitation. Temperature increase of sample solutions during USP laser treatments, as monitored by a thermocouple, did not exceed 2°C. The tightest focused spot of the laser beam was approximately 100 μm and the average laser exposure time of individual virion within the beam is estimated to be about 3.5 s.

All the experiments were carried out in triplicate. The errors were expressed in standard deviations (SD). Temperature was measured during laser-irradiation experiments with a thermocouple.

### Transmission electron microscopy

MNV-1 samples were visualized using 400 mesh, copper grids (Ted Pella Inc., Redding, CA) coated with a 0.25% Formvar solution (Electron Microscopy Sciences, Fort Washington, PA). Grids negatively stained with 1% phosphotungstic acid (Polysciences Inc., Warrington, PA), pH 7.0 and examined under a Philips CM12 transmission electron microscope (Philips, Eindhoven, The Netherlands) at an accelerating voltage of 80 KV. Images were collected with a 4000 M-T1-GE-AMT detector (DVC Co., Austin, TX) and processed with AMT V600 software (AMT, Danvers, MA).

### Real-Time PCR measurements

Real-Time PCR measurements were performed on the genome of MNV-1 without the laser irradiation (control) and after the laser irradiation at an average laser power density of 100 MW/cm^2^, following the standard procedures as described by the manufacturer, by using Applied Biosystems model 7500 fast Real-Time PCR system from life Technologies. For MNV-1, sense primer 6622 s 5′-cgcctttaccaattggcc-3′ and antisense primer 6875 5′-tgaaagagttggtttggagc-3′ were used at an annealing temperature of 64°C to produce a 273-bp amplicon [14].

Reverse transcription of all viral RNA was performed at 50°C for 30 min followed by a 15-min Taq activation step at 95°C. Forty cycle PCR reactions were performed using annealing times of 1 min, a 1-min extension step at 72°C and a 30-s denaturation step at 95°C. For the final cycle, the annealing time was extended to 2 min and the final extension was performed for 10 min. All primers were used at a final concentration of 0.1 μg/50 μl reaction mix or approximately 0.25 μM for each primer.

## Results

### Efficient inactivation of MNV-1 by USP laser irradiation

**Figure 1:**
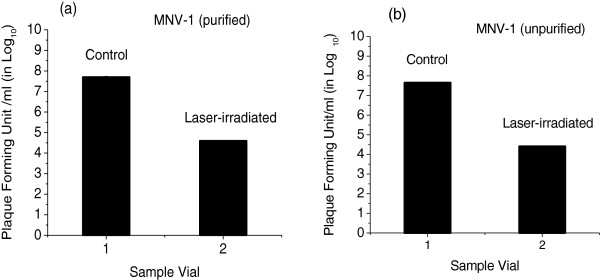
**Inactivation of MNV-1 by the visible ultrashort pulsed laser. (a)** Graphs of plaque forming units (PFU)/ml for control (without laser irradiation) and laser irradiated MNV-1 purified samples. **(b)** Graphs of plaque forming units (PFU)/ml for control (without laser irradiation) and laser irradiated MNV-1 unpurified samples. The USP laser is operated at a wavelength of 425 nm with a pulse width of 100 fs, a repetition rate of 80 MHz, and an average power of 120 mWs. The laser exposure time was 2 h. S.D error bars are small to be readily observed.

Since virus stocks and samples are often propagated in complex tissue culture media that contains potentially chromogenic constituents such as neutral red indicator dye, amino acids, cellular proteins and nucleic acids, MNV-1 used in this work was propagated in indicator-free media and partially purified by sucrose gradient or Optiprep gradient ultracentrifugation, respectively. Figure 1(a) shows a bar graph of the plaque forming units (PFU) for control and laser-irradiated MNV-1 samples in buffer solution. The control represents the sample with no laser irradiation. We observed a load reduction of 3.1 ± 0.1 in log_10_ scale for the laser-treated MNV-1 group. Here, load reduction is defined as the ratio of the number of plaque in the control to the number of plaque in the laser-irradiated sample. For reference, Figure 1(b) shows the load reduction of 3.0 ± 0.1 in log_10_ scale for the unpurified laser-treated MNV-1.

We observed that the temperature of laser-treated samples did not increase by more than 2°C above room temperature as monitored by a thermocouple.

### Laser power-density dependence of inactivation of MNV-1

**Figure 2:**
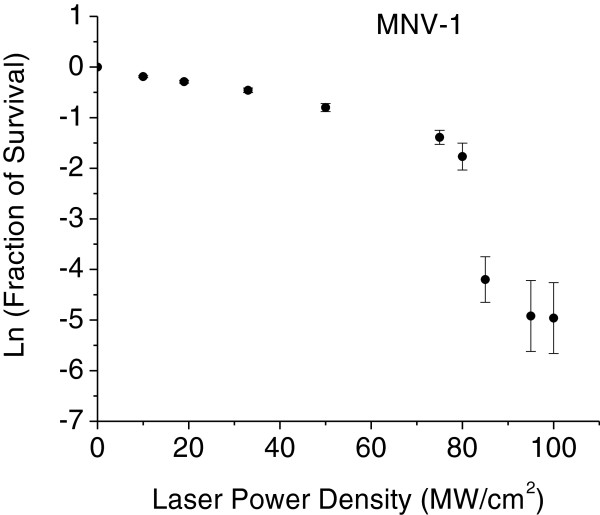
**The fraction of survival as a function of laser power density for a MNV-1 sample.** A rapid decrease of the fraction of survival has been found at a laser power density of around 80 *MW*/*cm*^2^.

To gain better insight into the mechanism of inactivation for these non-enveloped, icosahedral viruses by USP laser irradiation, we measured the fraction of MNV-1 survival as a function of laser power density, which is shown in Figure 2 in natural logarithm (ln) scale. The laser exposure time was 2 hours. Here, the fraction of survival is defined as the reciprocal of the load reduction. The fraction of virus survival has been found to decrease as the laser power density increases in a continuous fashion up to about 80 MW/cm^2^. As the laser power density increases beyond 80 MW/cm^2^, the fraction of survival drops precipitously.

### Transmission electron microscope images of laser-irradiated MNV-1

**Figure 3:**
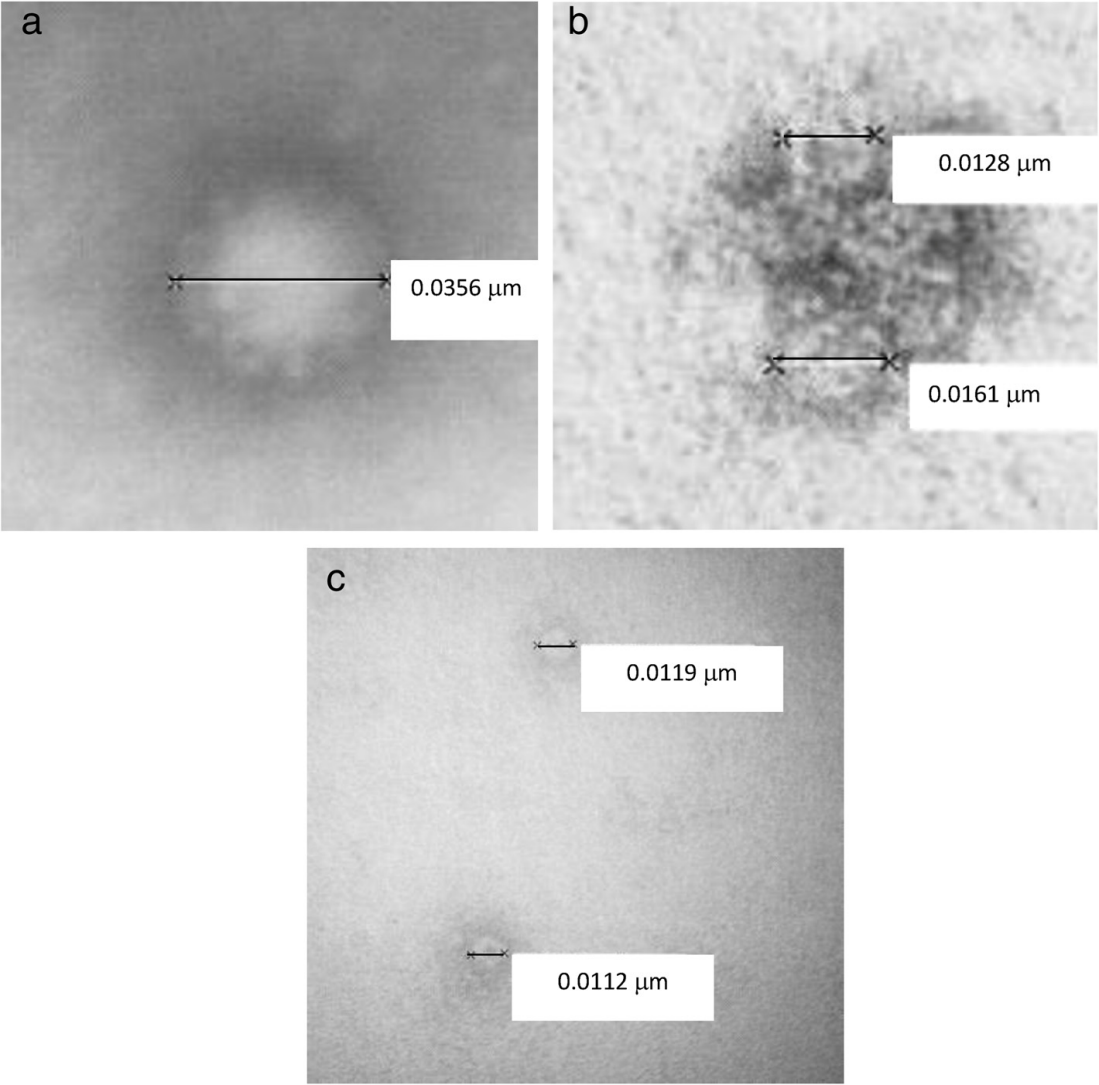
**Typical TEM images of MNV-1. (a)**: without laser irradiation (the control); **(b)**: with laser irradiation at a power density of 1.1 ± 0.2 *MW*/*cm*^2^ (here, for the sake of clarity, only one inactivated MNV-1 is shown); **(c)**: with laser irradiation at a power density of 100 ± 10 *MW*/*cm*^2^. The spherical structure with a diameter of around 30 nm in **(a)** represents the presence of MNV-1 in the control. At the intermediate laser power density, **(b)** shows that the inactivated MNV-1 particle forms cracks at the structural links of the capsid. At the high laser power density, **(c)** demonstrates the disintegration of the capsid of the inactivated MNV-1 into spherical structures with a diameter of around 10 nm.

Typical transmission electron micrographs of the control and laser-irradiated MNV-1 samples are shown in Figure 3. The spherical structure of diameter about 30 nm in Figure 3(a) shows the presence a MNV-1 particle in the control. Figure 3(b) shows that, after laser irradiation with a power density of 1.1 ± 0.2 *MW/cm*^2^, the capsid of the inactivated MNV-1 becomes cracked, presumably along the weak structural links, but remains intact, as evidenced by the appearance of smaller structures of about 10 nm in diameter on the capsid (here, for the sake of clarity, only one inactivated MNV-1 is shown). This transmission electron microscope image clearly reveals the locations of weak structural links on the capsid of a non-enveloped, icosahedral virus – MNV-1. Figure 3(c) shows that as the laser power density increases to 100 ± 10 *MW/cm*^2^, the capsid of the inactivated MNV-1 becomes disintegrated and separated into small pieces of diameter about 10 nm.

### USP laser spectral-width dependence of inactivation of MNV-1

**Figure 4:**
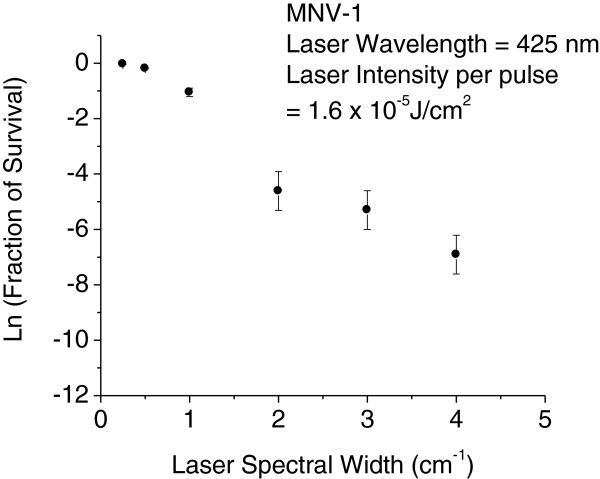
**MNV-1 survival as a function of laser spectral width.** The laser intensity is kept constant as shown. The fraction of survival decreases rapidly at a laser spectral width of around 1.0 *cm*^-1^, is consistent with the prediction by the impulsive stimulated Raman scattering process using a one laser configuration. Laser exposure time was 2 h. Error bars represent S.D.

The theory of ISRS developed for the single-pulse excitation configuration makes predictions that the energy contained within the spectral width of a USP laser must be larger than the energy of a given molecular vibration to excite that specific vibrational motion in the molecule [15]. In order to test whether the inactivation is due to an ISRS process or not, inactivation experiments were also carried out as a function of the laser spectral width. The results are shown in Figure 4. Virus inactivation has been found to be very sensitive to the FWHM of the laser spectral width. Very limited or no inactivation was observed for laser spectral width ≤ 1 cm^-1^, while substantial inactivation was observed for laser spectral width ≥ 2 cm^-1^.

### Real-Time PCR results on the genome of MNV-1 without and with USP laser irradiation

**Figure 5:**
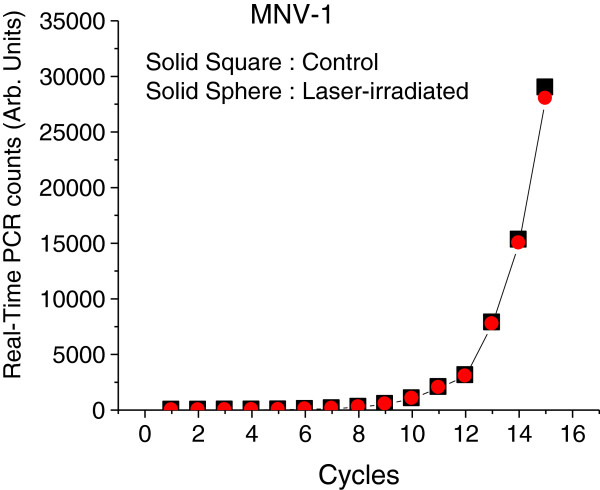
**Real-time PCR counts in linear scale for the genome of MNV-1 without (control) and with laser irradiation at an average laser power density of 100 MW/cm**^**2**^**.** The similarity of real-time PCR counts between the control and laser-irradiated samples suggests that there is very minimal genome degradation/damage for MNV-1 after the laser irradiation.

In order to test the effects of USP laser irradiation on the genome of MNV-1, Figure 5 shows the real-time PCR counts in linear scale for the genome of MNV-1 without (control) and with laser irradiation at an average laser power density of 100 MW/cm^2^. The similarity of real-time PCR counts between the control and laser-irradiated samples suggests that, within the amplicon size of 273 bp tested, there is very minimal genome degradation/damage for MNV-1 after the laser irradiation. This result is consistent with our previous reports by using gel electrophoresis that USP laser irradiation under similar experimental conditions does not damage the genome of either M13 bacteriophage [6,8] or MCMV [10].

## Discussion

As mentioned previously, potential mechanisms for the inactivation of non-enveloped, icosahedral viruses such as MNV-1 include: (i) production of UVC photons by non-linear optical process; (ii) thermal heating by the direct laser absorption; (iii) one-photon absorption process by chromophores, such as porphyrin molecules [16] or indicator dyes producing reactive oxygen species, or other reactive species; and (iv) ISRS process. We now examine these possible mechanisms in detail.

### Inactivation cannot be due to generation of UVC photons through non-linear optical processes

First, we have estimated the number of UVC photons which might be produced at laser power densities used in our experiments. The non-linear optical coefficient for murine norovirus is not available in the literature; however, if we assume that the viruses are giant molecules and that their non-linear coefficients are comparable to that of a typical molecule [17], then the number of UVC photons generated under our experimental conditions is estimated to be of the order of 0.1 per second. Because the laser exposure time is two hours, the titer of viral particles is about 50 million; the value of generated UVC photons is too small to account for the load reduction observed in our inactivation experiments for MNV-1.

Secondly, we have tried to detect the UVC photons which might be generated in our laser experiments (here, the glass vial is replaced by a synthetic fused silica vial) by using a UV spectrometer with a photon counting system. Within our experimental uncertainty of ±1photon per second we failed to detect any of the UVC photons. This finding is consistent with our estimation stated above. Therefore, the observed inactivation for MNV-1 cannot be due to UVC photons generated by non-linear optical process.

### Inactivation is not due to thermal heating effects

In order for the USP laser to thermally damage the virus, there must be direct energy transfer between the laser and viral particle. We observed that the temperature of laser-treated samples did not increase by more than 2°C above room temperature as monitored by a thermocouple. MNV-1 is known to be highly thermostable, and readily tolerant of temperatures in excess of 60°C [18,19]. Therefore, heating of the entire sample, termed macro-thermal effects, can be ruled out as an explanation for inactivation.

However, we note that it is possible that laser treatment could create “micro-thermal” effects where the laser energy might be absorbed by the viral particle itself and spread throughout the volume of the virus, heating up the viral particle and leading to the inactivation. In this micro-thermal scenario, one would expect that for a given laser power density, viruses with different sizes would be heated to different temperatures.

For icosahedral viruses, if we consider a simple model calculation in which the capsid of the virus is assumed to completely absorb the incident photons and the energy thermalization within the viral particle is significantly faster than that between the viral particle and its surrounding, the total deposited energy is expected to be proportional to the cross-sectional area of the virus. In the micro-heating scenario, this deposited energy is to spread/thermalize over the whole volume of virus and increase the temperature of the virus.

The total laser energy deposited is given by

(1) E=I∗S=m∗c∗∆T=ρ∗V∗c∗∆T;

where *I* is the laser intensity.

*S* is the cross-sectional area of the virus; *m* is the mass of the virus; *c* is the specific heat of the virus; ∆*T* is the increase of temperature of the virus; *ρ* is the density of the virus; *V* is the volume of the virus.

For a given laser intensity *I*, if *ρ*,*c* are assumed to be constant, because *S* ∝ *r*^2^ and *V* ∝ *r*^3^, we have

(2) $$∆T\propto \frac{1}{r};$$

where *r* is the radius of the virus.

Therefore, for a given laser intensity *I*, the expected temperature increase (∆*T*) would be less for a larger virus than for a smaller virus.

When we compare load reduction of MNV-1 and HPV -16 experiments under the same laser parameters, we find that the load reduction of 3.1 ± 0.1and 5.1 ± 0.5 in log_10_ scale for MNV-1, HPV-16 (which is not shown here), respectively. The diameter for MNV-1 and the HPV-16 pseudovirion is approximately 30 and 55 nm, respectively. Thus, the observation that the larger icosahedral virus is more readily inactivated, argues against a micro-thermal mechanism. We note that even though the energy transfer between the virion and its environment is taken into account, because a larger virus has a larger surface area, resulting in greater energy loss, and thereby a lesser temperature increase (∆*T*), which again is in contradiction with our experimental observation of load reductions for MNV-1 and HPV-16.

### Inactivation is not due to one-photon absorption

For this one-photon absorption scenario in which chromophores present in the sample solution, upon absorption of laser wavelength at 425 nm, may produce reactive oxygen species, leading to the inactivation of viral particles. This possibility is unlikely for the following reasons: (1) We have not observed any detectable absorption by MNV-1 viral particles at a wavelength of 425 nm; (2) MNV-1 used in this work were propagated in indicator-free media and substantially purified by sucrose gradient and Optiprep gradient centrifugation respectively, which presumably removed extraneous cellular proteins which might absorb blue laser light; (3) we have tested and found that the load reduction for the purified MNV-1 samples was consistent with that for which MNV-1 samples were not purified; (4) we tested the inactivation of MNV-1 by the USP laser with the orange juice as well as apple juice spiked with MNV-1. Within the experimental uncertainty, the load reduction of MNV-1 in MNV-1-spiked orange/apple juice was found to be the same as that in the buffer solution. Because both orange juice and apple juice are loaded with antioxidant – vitamin C, which is able to efficiently scavenges reactive oxygen species, these experimental results further confirm that the inactivation of MNV-1 by the USP laser cannot be due to the production of reactive oxygen species through the one-photon absorption process.

### Our experimental results are consistent with inactivation by an ISRS process

ISRS has been shown to be able to excite vibrational oscillations in solids and molecules [15,20-26]. In general, these experiments have been performed by using two independently-tunable, USP lasers emitting separate and distinct wavelengths. However, in this work, we employed a special one-laser-beam excitation configuration originally described by Yan et al. [15]. The theory of the ISRS process under a one-laser-beam excitation configuration shows [15,27] that when the laser intensity is kept constant, the amplitude of the laser excited vibrational motion is proportional to $e^{-\left({\omega_{0}}^{2}/∆{\omega_{L}}^{2}\right)/4}$, where *ω*_0_ is the angular frequency of the excited vibrational motion and *∆ω*_*L*_ is the full-width-at-half-maximum (FWHM) of the laser spectral width. Because the excited amplitude of vibration depends on the factor – $\left({\omega_{0}}^{2}/∆{\omega_{L}}^{2}\right)/4$ in an exponential fashion, the ISRS process predicts that a threshold laser spectral width, which is comparable to the angular frequency of the global vibrational motion of the virus exists, for viral inactivation. Specifically, because the angular frequency of the global vibrational modes of an icosahedral virus with 30 nm in diameter is estimated to be about 2 cm^-1^[28], the ISRS inactivation mechanism predicts that there exists a laser spectral-width threshold of about 1 cm^-1^ for the inactivation of MNV-1 by a USP laser. This prediction is indeed observed in our experiments as described below.

To verify this prediction by ISRS inactivation mechanism, we have performed the inactivation experiments for MNV-1 as a function of the laser spectral width, which is shown in Figure 5. If we take *ω*_0_ to be 2.0 *cm*^-1^ for a non-enveloped, icosahedral virus with a diameter of 30 nm (28), $e^{-\left({\omega_{0}}^{2}/∆{\omega_{L}}^{2}\right)/4}$ has values of 8.9 *×* 10^-6^, 1.8 *×* 10^-2^, 3.7 *×* 10^-1^, 7.7 *×* 10^-1^, 8.9 *×* 10^-1^, 9.4 *×* 10^-1^ for ∆*ω*_*L*_ = 0.25, 0.50, 1.0, 2.0, 3.0, 4.0 *cm*^-1^, respectively. In other words, the ISRS process predicts that in order to excite a sizable vibrational amplitude to break hydrogen bonds/hydrophobic contacts and achieve inactivation, the laser spectral width has to be comparable to, or larger than, the angular frequency of the oscillations. This prediction is indeed in consistence with the experimental results of Figure 5 in which very little or no inactivation has been found when the laser spectral width ∆*ω*_*L*_ is ≤ 0.5 *cm*^-1^; while significant inactivation is found for *∆ω*_*L*_ ≥ 2 *cm*^- 1^. Consequently, we attribute the inactivation mechanism of the non-enveloped, icosahedral MNV-1 by the USP laser to the ISRS process.

**Figure 6:**
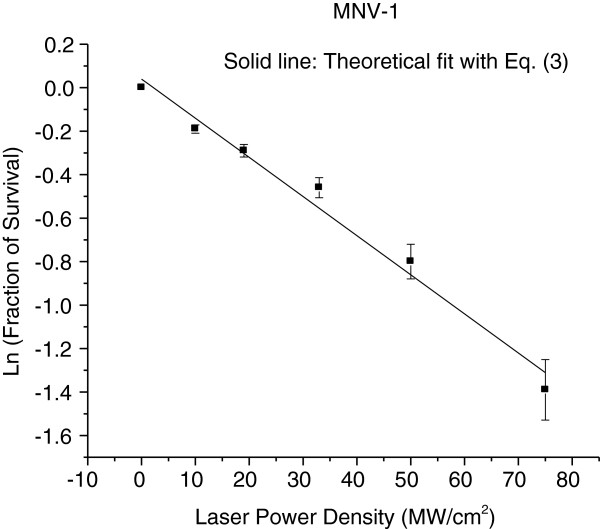
**The MNV-1 survival fraction as a function of laser power density is fit with Eq. (3) for laser power density < 80 *MW*/*cm***^**2 **^**(solid line).** Error bars represent S.D.

To explain the data for the USP laser inactivation of MNV-1 in Figure 2, we divided the data into two parts: the first part consisted of data prior to the occurrence of the sharp-drop of the fraction of survical (i.e., for laser power density up to 75 MW/cm^2^) and the second part consisted of data within the observed sharp-drop of the fraction of survival (power density ≥ 80 MW/cm^2^). We have found that the first part of the data can be fit pretty well by the following function [29]:

(3) $$y=\exp\left(-\mathrm{Ax}\right);$$

where *y* is the fraction of survival; *x* is the laser power density and A is the effective inactivation rate constant. Figure 6 shows the best fit of our data, modeled as a solid line with *A* = (0.017 ± 0.001)*cm*^2^/*W*.

We did not attempt to fit the second part of the data with laser power density ≥ 80 MW/cm^2^. However, the qualitative behavior – sharp drop of the fraction of survival in this range of laser power densities, indicated that the effective inactivation rate in this range of laser power densities is much larger than that obtained from the first part of the data with laser power density up to 75 MW/cm^2^.

Considering the energy required to break hydrophobic contacts and hydrogen bonds, it is possible to explain the observed laser power density results shown in Figures 2 and 6. Two disparate inactivation curves were observed; one for laser power densities up to 75 MW/cm^2^ and the other for laser power densities of 80 MW/cm^2^ and above. We note that the energy of a typical covalent bond in a biological system is about 4 eV or larger. In our laser experiments, the relatively low photon energy of 2.92 eV generated from the USP laser employed at a wavelength of 425 nm means that weaker hydrogen bonds or hydrophobic contacts can be broken but not the stronger covalent/ionic bonds. Furthermore, the broken hydrogen bonds and hydrophobic contacts in a molecule have been demonstrated to reform on a time scale of about 10 picoseconds at room temperature (30, 31). With this knowledge in mind, we are able to explain the relatively small effective inactivation rate constant A (from equation (3)) deduced for laser power density of ≤ 75 MW/cm^2^. Because the laser power density is relatively small, the number of broken hydrogen bonds/hydrophobic contacts is relatively small; and since these broken bonds are rapidly reformed [30,31], the majority of the laser-irradiated viruses having broken bonds is expected to have their broken bonds reformed and restored to their non-irradiated structure. The capsid of a virus is an integral part in the viral infection process. Any damage/alternation to its structure such as breaking of some hydrogen bonds or hydrophobic contacts can significantly affect its infection capability. Therefore, it is the minority of the laser-irradiated viruses which has not restored to their original structure that contributes to the relatively smaller effective inactivation rate constant observed in this range of laser power densities. The laser power density of 75 MW/cm^2^ simply reflects the threshold laser power density in which the number of broken bonds that can be easily reformed and the structure of virus can be easily restored for MNV-1. On the other hand, at the laser power density of ≥ 80 MW/cm^2^, inactivation for MNV-1 was dramatically enhanced, as indicated by the sharp-drop of the fraction of survival in this range of laser power density. We interpret this to be reminiscence of a deposited laser energy threshold through the ISRS process that once exceeded, results in so many simultaneously broken hydrogen bonds and hydrophobic contacts that the entire capsid is disintegrated spatially (as shown in TEM image of Figure 3(c)), as a result reformation of bonds and restoration to the original viral structure is greatly reduced. Therefore, at the laser power density of ≥ 80 MW/cm^2^, the capsid of MNV-1 begins to disassemble and the effective inactivation rate increases dramatically. It is worthwhile mentioning that the TEM images shown in Figure 3 not only provide direct experimental evidence of the disruption of the capsid in a non-enveloped, icosahedral virus by USP laser irradiation but also reveal the locations of weak structural links on its capsid. This paramount information can help understand the fundamental structure of non-enveloped, icosahedral viruses.

We note that in the previous studies of inactivation of enveloped virus like MCMV [10], the global structure of the non-irradiated and USP laser-inactivated viruses are almost identical. However, the protein gel electrophoresis experiments indicated that the inactivation was due to protein aggregation within the virion, induced by the ISRS process. The aggregate was identified to be composed of capsid protein and tegument protein by mass spectrometry analysis. For MCMV, the icosahedral nucleocapsid is surrounded by tegument protein and a lipid envelope through which a number of glycoproteins protrude. We think the reason why the MCMV capsid remained intact after the USP laser inactivation is most likely because of significant damping produced on the capsid of MCMV by the surrounding amorphous tegument and lipid envelope layers.

## Conclusion

We have investigated the inactivation mechanism of non-enveloped, icosahedral viruses - MNV-1 by USP laser irradiation. Possible mechanisms of inactivation are thoroughly examined. Real-time PCR measurements indicate that, within the amplicon size of 273 bp tested, USP laser irradiation does not degrade the genome of MNV-1. We conclude that our model non-enveloped virus, MNV-1, is inactivated by the ISRS process. These studies provide fundamental knowledge on photon-virus interactions on femtosecond time scales. From the analysis of the TEM images of viral particles before and after USP laser irradiation, the locations of weak structural links on the capsid of MNV-1 were revealed. This important information will greatly aid our understanding of the structure of non-enveloped, icosahedral viruses. We envision that this efficient, non-invasive approach for the eradication of non-enveloped, icosahedral viruses, when employed in a continuous, syringe-pumped configuration, can be applied to the disinfection of pathogens in the pharmaceutical processes and disinfection of blood components for blood transfusion.

## Competing interests

The authors declare that they have no competing interests.

## Authors’ contributions

SWDT plans and carries out the laser experiments, performs the PCR experiments, explains the experimental results, writes the manuscript; DHK prepares the virus samples, performs the PCR experiments, carries out the assays, helps explain the experimental results and writes the manuscript. CP carries out the laser experiments. SA plans the laser experiments, explains the experimental results. DSS performs the TEM experiments. TCW prepares the virus samples, carries out the assays. KTT plans and carries out the laser experiments, explains the experimental results and writes the manuscript. All authors read and approved the final manuscript.
